# 1526. Hospitalized Patients with HIV – Defining the Population and the Impact of Barriers to Care

**DOI:** 10.1093/ofid/ofad500.1361

**Published:** 2023-11-27

**Authors:** Timothy Hatlen, Giselle A Reynoso, Kirk B Fetters

**Affiliations:** Harbor-UCLA Medical Center, Torrance, California; Lundquist Institute at Harbor-UCLA Medical Center, Torrance, California; Harbor-UCLA Medical Center, Torrance, California

## Abstract

**Background:**

Hospitalized people living with HIV (PLWH) with active viremia present a unique opportunity for addressing barriers to care (BTC) and optimizing 1-year outcomes. Large-scale interventions have had limited long-term success. We aimed to evaluate the role of pre-specified BTC to viral suppression at one year to guide local interventions.

**Methods:**

We performed a retrospective cohort study at a safety-net hospital in Los Angeles County evaluating encounters of adult PLWH admitted between 2015 - 2020. We collected demographics, clinical characteristics, and 1-year outcomes based off baseline virologic status.

**Results:**

A total of 361 encounters involving 234 patients were reviewed. Patients viremic at baseline (n=70) were more often Black, cis-men, severely immunocompromised and 73% reported at least one BTC (Table 1, Fig 1). At 1-year follow up, patients with baseline viremia or reporting multiple BTC regardless of baseline viral status were more likely to be viremic or lost to follow up (Table 2, Fig. 2).

Population characteristics based off insurance status and baseline virologic status

**Figure:**
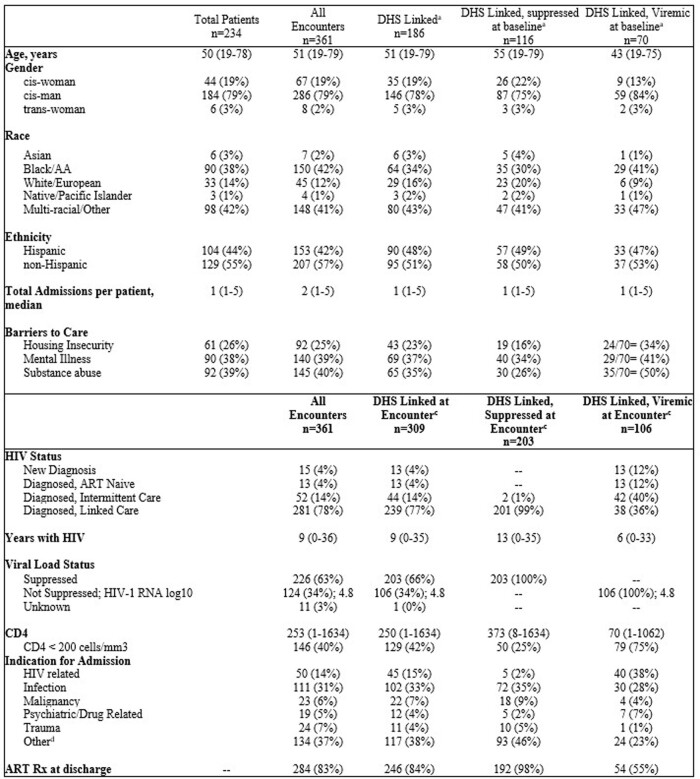

Data are n (%) or median (range). Categories may not add to 100% in cases of missing data. a – Based off first encounter; b - Encounters of patients with insurance though Department of Health Services or self-pay that have ability to follow up in the safety-net system included only; c – includes data for all patient encounters; d – other diagnoses include, but not limited to, chest pain, acute coronary syndrome, heart failure, diabetes with hyperglycemia, cerebrovascular event; c – excluding patients that died or discharged on hospice care; Suppressed – HIV-1 RNA < 200 copies, ART – antiretroviral therapy; dx - diagnosis

One Year Outcomes based off Baseline Viral Status in the Insurance Linked Population

**Figure:**
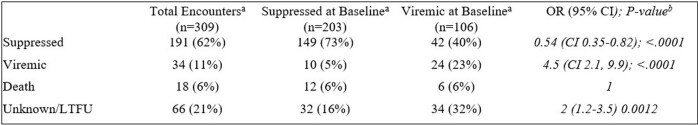

a – Encounters of patients with insurance though Department of Health Services or self-pay that have ability to follow up in the safety-net system included only; b – Chi Square suppressed versus viremic baseline; LTFU – lost to follow up; Suppressed – HIV-1 RNA < 200 copies/mm3. Viremic – HIV-1 RNA > 200 copies/mm3

Prevalence of barriers to care amongst PLWH suppressed or viremic at baseline

**Figure:**
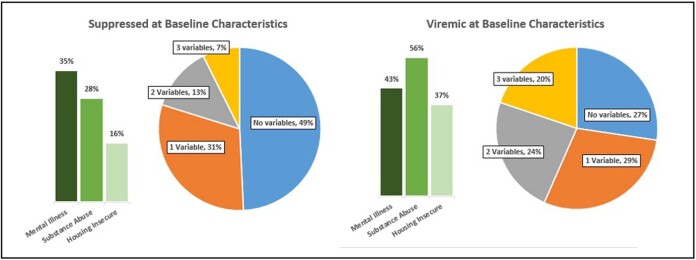

**Conclusion:**

Hospitalized PLWH experiencing multiple barriers to care, regardless of baseline virologic control, are at higher risk for being viremic at 1-year and lost to follow up. These data can inform local interventions, i.e. enhanced post-discharge follow-up or social work involvement, and advocacy in this population.

**Figure:**
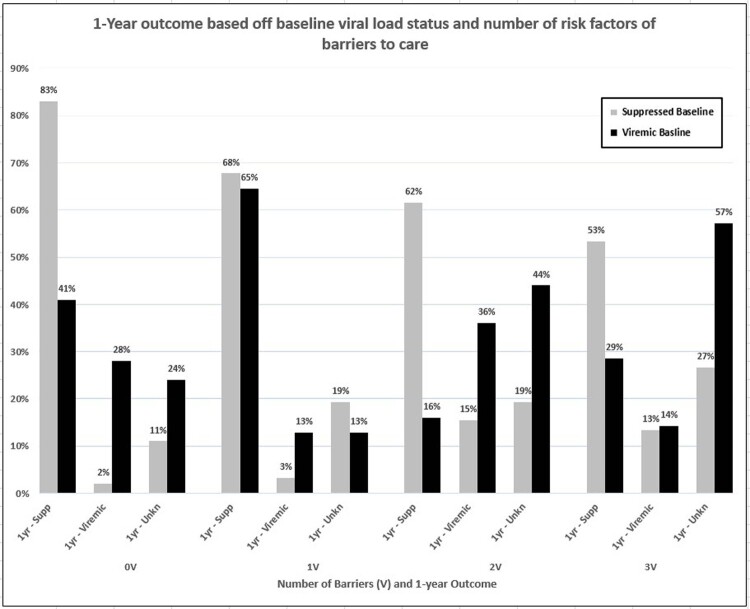

LTFU – lost to follow up; 1yr – 1-year; #V – cumulative number of prespecified barriers to care. Suppressed Baseline defined as HIV-1 RNA < 200 copies/mm3

**Disclosures:**

**All Authors**: No reported disclosures

